# Gene expression and DNA methylation as mechanisms of disturbed metabolism in offspring after exposure to a prenatal HF diet[S]

**DOI:** 10.1194/jlr.M092593

**Published:** 2019-05-07

**Authors:** Sven H. Rouschop, Tanja Karl, Angela Risch, Petronella A. van Ewijk, Vera B. Schrauwen-Hinderling, Antoon Opperhuizen, Frederik J. van Schooten, Roger W. Godschalk

**Affiliations:** Department of Pharmacology and Toxicology,* NUTRIM School of Nutrition and Translational Research in Metabolism, Maastricht University, Maastricht, The Netherlands; Department of Biosciences† University of Salzburg, Salzburg, Austria; Department of Radiology and Nuclear Medicine§ Maastricht University Medical Center, Maastricht, The Netherlands; Netherlands Food and Consumer Product Safety Authority (NVWA),** Utrecht, The Netherlands

**Keywords:** obesity, pregnancy, liver, microarrays, diet and dietary lipids, in utero, epigenetics, lipid metabolism, oxidative stress, development, deoxyribonucleic acid, high-fat diet

## Abstract

Exposure to a prenatal high-fat (HF) diet leads to an impaired metabolic phenotype in mouse offspring. The underlying mechanisms, however, are not yet fully understood. Therefore, this study investigated whether the impaired metabolic phenotype may be mediated through altered hepatic DNA methylation and gene expression. We showed that exposure to a prenatal HF diet altered the offspring’s hepatic gene expression of pathways involved in lipid synthesis and uptake (SREBP), oxidative stress response [nuclear factor (erythroid-derived 2)-like 2 (Nrf2)], and cell proliferation. The downregulation of the SREBP pathway related to previously reported decreased hepatic lipid uptake and postprandial hypertriglyceridemia in the offspring exposed to the prenatal HF diet. The upregulation of the Nrf2 pathway was associated with increased oxidative stress levels in offspring livers. The prenatal HF diet also induced hypermethylation of transcription factor (TF) binding sites upstream of lipin 1 (*Lpin1*), a gene involved in lipid metabolism. Furthermore, DNA methylation of *Lpin1* TF binding sites correlated with mRNA expression of *Lpin1*. These findings suggest that the effect of a prenatal HF diet on the adult offspring’s metabolic phenotype are regulated by changes in hepatic gene expression and DNA methylation.

The parents’ diet before and during pregnancy significantly determines their offspring’s risk of developing diseases later in life (1–7). Observational studies have shown that maternal consumption of a high-fat (HF) diet and obesity during pregnancy and lactation predispose offspring to developing various metabolic disorders, such as obesity and type 2 diabetes, and cardiovascular diseases (8–11). In mice, a maternal HF diet leads to increased body weight and fat mass and decreased energy expenditure in offspring (12, 13). Similarly, a paternal HF diet before conception is able to increase the offspring’s bodyweight and fat mass and impair its lipid and glucose metabolism (14, 15). In addition, both maternal and paternal HF diet have been shown to induce nonalcoholic fatty liver disease in mouse offspring, indicated by increased liver size, excessive accumulation of cholesterol and triglycerides (TGs), and eventually hepatic inflammation (13, 14). Moreover, reports studying the influence of both paternal and maternal diet showed that the combination of these diets has an additive effect on mouse offspring health, leading to more prominent negative effects in offspring compared with only a paternal or maternal HF diet (14, 16).

Although the effects of a prenatal HF diet on the offspring phenotype have been studied thoroughly, it is less known through which mechanisms these long-lasting effects are transmitted. The responsible molecular mechanisms for this have been suggested to involve epigenetics, because both human and animal studies demonstrated that prenatal exposures alter DNA methylation in the offspring (12, 13, 17, 18). In addition, both maternal and paternal HF diets have been shown to modify gene expression, predominantly affecting pathways related to metabolism and inflammation (12, 13, 17). Altering these metabolic and inflammatory pathways contributed to the impaired metabolic phenotype in the offspring of HF diet-fed mice. Altogether, this leads to the hypothesis that a prenatal HF diet changes DNA methylation in offspring, which subsequently alters gene expression, thereby affecting the offspring’s phenotype.

To test this hypothesis, we studied the effect of a combined paternal and maternal HF diet on hepatic DNA methylation and gene expression in mouse offspring. In this study, both parent mice received a HF or low-fat (LF) diet before and during pregnancy and throughout lactation, after which offspring were weaned onto a HF diet. Feeding both parents the same diet and weaning offspring onto a HF diet better approximates the current Western situation in which the diet is characterized by a HF content, and eating patterns are shared by individuals from the same family (19). Only male offspring were included for analyses, because sex differences are known to occur in the response to a prenatal HF diet (12, 20–23). Previous work showed that, at 12 weeks of age, the offspring displayed postprandial hypertriglyceridemia, which related to an impaired clearance of lipids from the blood into the liver after a HF meal (24).

To study the mechanisms underlying the impaired metabolic phenotype after prenatal exposure to a HF diet, hepatic genome-wide gene expression was studied using whole transcript microarrays. In addition, hepatic DNA methylation of selected genes was assessed by bisulfite pyrosequencing. This work shows that exposure to a prenatal HF diet alters gene expression of pathways involved in hepatic lipid synthesis and uptake, oxidative stress response, and cell proliferation. Furthermore, the prenatal HF diet induces hypermethylation of transcription factor (TF) binding sites upstream of lipin 1 (*Lpin1*), a differentially expressed gene involved in lipid metabolism.

## MATERIALS AND METHODS

### Study design and workflow

**Fig. 1.**
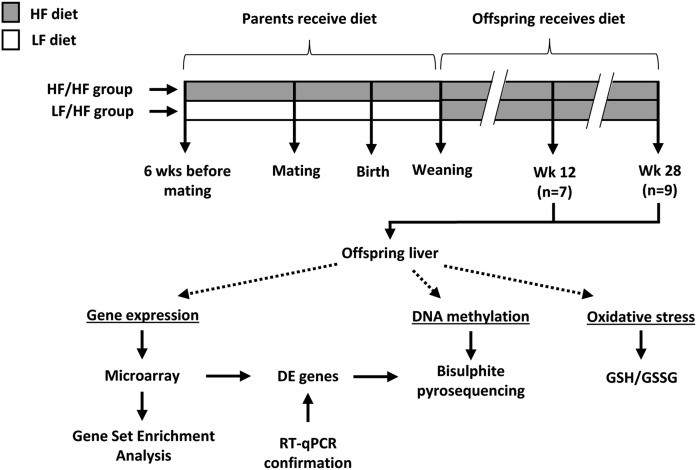
Overview of study design and workflow. Parent mice received a HF or LF diet starting 6 weeks before mating and continuing during pregnancy and lactation. Offspring were weaned onto a HF diet, thereby creating the experimental groups LF/HF and HF/HF, with the first two letters indicating the parental diet and the last two letters indicating the offspring diet. At 12 and 28 weeks of age, offspring livers were collected and analyzed. Gene expression was assessed using microarrays and GSEA. Selected differentially expressed (DE) genes were confirmed with real-time qPCR. DNA methylation of TF binding sites upstream of differentially expressed genes was measured using bisulfite pyrosequencing. Oxidative stress was measured with the GSH/GSSG assay.

A schematic overview of the study design and workflow is presented in **Fig. 1**.

### Animal model

Animal experiments were approved by the Institutional Ethics Committee on Animal Welfare of Maastricht University. Mice were housed under standard conditions at 25°C with a 12-12 h light-dark cycle with ad libitum access to water and feed. Specific pathogen-free C57Bl/6J (Charles River, Saint-Germain-Nuelles, France) parent mice were randomly assigned to either a HF diet [45% kcal fat (31% saturated, 36% monounsaturated, and 33% polyunsaturated fatty acids), 20% kcal protein, and 35% kcal carbohydrate; Research Diets, New Brunswick, NJ) or a standard chow LF diet (9% kcal fat, 33% kcal protein, and 58% kcal carbohydrate; ssniff Spezialdiäten GmbH, Soest, Germany). The diet started 6 weeks before mating and continued throughout gestation and lactation. Maternal food intake and bodyweight were measured weekly (24). At weaning, all offspring received a HF diet, thereby creating the experimental groups HF/HF and LF/HF, with the first two letters indicating the parental diet and the last two letters indicating the offspring diet. Offspring was weaned onto a HF diet, because this experimental setup provides a better representation of the Western diet, which is predominantly characterized by a HF content. Litter size and offspring food intake were similar for both experimental groups (24). Only male offspring were included for further experiments. Phenotypic outcomes in offspring between 12 and 28 weeks of age were previously reported (24). To study how these effects of the prenatal HF diet were transmitted, livers were obtained from the offspring at 12 weeks of age (n = 7 per group) and 28 weeks of age (n = 9 per group) and were used for subsequent measurements in gene expression, oxidative stress, and DNA methylation.

### Gene expression microarray

Total RNA from offspring livers was extracted using an RNeasy Mini kit (Qiagen, Venlo, The Netherlands). RNA concentration and purity were assessed using a Nano Drop ND-1000 spectrophotometer (Isogen, IJsselstein, The Netherlands). RNA quality was measured on an Agilent 2100 bioanalyzer (Agilent Technologies, Amsterdam, The Netherlands). All RNA samples had an RNA integrity number >8.0. RNA (100 ng) was labeled with whole transcript sense target assay and hybridized to Affymetrix Mouse Gene 1.1 ST arrays (Affymetrix, Santa Clara, CA). Microarrays were analyzed using MADMAX pipeline for statistical analysis of microarray data (25). Signal intensities were normalized with the robust multichip average method and probes were annotated (26). Microarray data are available through the Gene Expression Omnibus database (accession number GSE123009).

### Gene set enrichment analysis

Functional data analysis of microarray data was performed with gene set enrichment analysis (GSEA) (27). GSEA focuses on gene sets (groups of genes that share common biological function or regulation) rather than individual genes. For GSEA, annotated gene sets were used from the databases, BioCarta (28), Kyoto Encyclopedia of Genes and Genomes (KEGG) (29), Reactome (30), and WikiPathways (31). Significantly affected gene sets were selected on a false discovery rate (FDR) q-value of <0.05 and were ranked by the normalized enrichment score (NES).

### Selection of candidate genes for DNA methylation analysis

Individual genes from the microarray were defined as differentially expressed when comparison of the normalized signal intensities showed a *P*-value <0.05 in two-tailed paired intensity-based moderated t-statistics and an absolute fold-change >1.2 (32). Differentially expressed genes were ranked by fold-change (HF/HF versus LF/HF at week 12) and filtered for involvement in lipid metabolism or inflammation. A selection was made for the top two genes that were differentially expressed at both week 12 and week 28 [cluster of differentiation 163 (*Cd163*) and HMG-CoA reductase (*Hmgcr*)] and the top five genes that were differentially expressed at only week 12 and not at week 28 [acetoacetyl-CoA synthetase (*Aacs*), *Lpin1*, phospholipase A2 group XVI (*Pla2g16*), serum amyloid A1 (*Saa1*), and interleukin 1 receptor type I (*Il1r1*)].

### Gene expression real-time quantitative PCR

To confirm differential expression of candidate genes for DNA methylation, real-time quantitative (q)PCR was used. First, RNA was reverse transcribed into cDNA using iScript (Bio-Rad, Veenendaal, The Netherlands). Real-time qPCR was performed on a Bio-Rad CFX384 real-time PCR system using iQ SYBR Green Supermix (Bio-Rad). Primer sequences (supplemental Table S1) were obtained from PrimerBank (33) and qPrimerDepot (34). Housekeeping genes, *Gapdh* and *Actb*, were used for normalization. Relative expression levels were quantified with the ΔΔCt method (35).

### Selection of TF binding regions

**Fig. 2.**
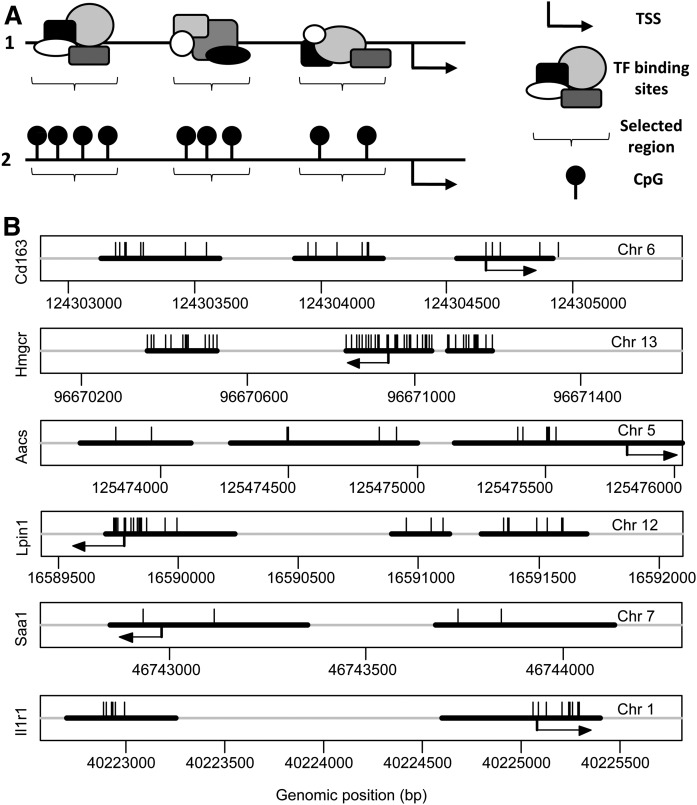
Identification of TF binding regions and selection of CpG sites for pyrosequencing. A1: Regions with a high density of TF binding sites were identified <2,000 bp upstream of the TSS of a selected gene, based on GTRD. A2: CpG sites within the selected TF binding regions were selected for DNA methylation analysis. B: Genomic positions of TF binding regions (indicated by black horizontal lines) and CpGs within these regions (indicated by vertical lines), which were selected for DNA methylation analysis. Arrows mark TSS.

Candidate genes of which differential expression was confirmed with real-time qPCR were used for subsequent DNA methylation analysis. For this purpose, CpGs were selected with the potential to regulate expression of the genes, *Cd163*, *Hmgcr*, *Aacs*, *Lpin1*, *Saa1*, and *Il1r1*. Regions with a high density of TF binding sites were identified <2,000 bp upstream of the transcription start site (TSS) of each gene (**Fig. 2A**) by using the Gene Transcription Regulation Database (GTRD; version 17.04) (36). GTRD is a database of TF binding sites identified by ChIP-seq experiments (36). Genomic locations of the selected TF binding regions and CpGs within those regions are shown in Fig. 2B. Methylation of CpG sites within the identified TF binding regions could potentially influence TF binding and thus regulate gene transcription. Therefore, methylation of these CpGs was measured using pyrosequencing.

### Pyrosequencing

To assess DNA methylation levels of selected CpG sites, a pyrosequencing approach was used. For this, DNA from offspring livers was extracted using the DNeasy blood and tissue kit (Qiagen) and subjected to bisulfite conversion with an EZ DNA methylation kit (Zymo Research, Irvine, CA) and subsequently used as a template for amplification of the selected TF binding regions. PCR was performed on an Eppendorf Mastercycler nexus (Eppendorf, Nijmegen, The Netherlands) using HotStarTaq DNA polymerase (Qiagen) and dNTP mix (Thermo Fisher Scientific, Eindhoven, The Netherlands). Biotin-labeled amplicons were captured with Streptavidin Sepharose beads (GE Healthcare, Vienna, Austria) and denatured and annealed to the sequencing primer using the PyroMark Q24 vacuum workstation (Qiagen). Amplicons were sequenced on a PyroMark Q24 (Qiagen), using PyroMark Q24 Advanced CpG reagents (Qiagen). Methylation of each CpG was quantitatively assessed using the PyroMark Q24 Advanced software. Assays and primers for PCR and pyrosequencing (supplemental Table S2) were designed with PyroMark Assay Design software.

### GSH and GSSG assay

To assess oxidative stress in offspring livers, GSH and GSSG were measured according to Rahman, Code, and Biswas (37). In short, liver homogenate was mixed with 5-sulfosalicylic acid to prevent further oxidation of GSH to GSSG. Supernatant of this mixture was used for both GSH and GSSG assessment. GSH was measured by oxidizing it with DTNB to form TNB, which is measurable with a spectrophotometer. For GSSG measurement, samples were first preincubated with 2-vinylpyridine to deplete all GSH. Subsequently, samples were incubated with NADPH and glutathione reductase, which reduces GSSG into GSH, and simultaneously incubated with DTNB to quantify the amount of GSH through TNB formation. GSH and GSSG concentrations were normalized to the starting amount of liver tissue.

### Statistical analysis

For GSH, GSH/GSSG ratio, real-time qPCR, and DNA methylation, differences between groups were tested by two-tailed independent samples Student’s *t*-test. For DNA methylation, *P*-values were corrected for multiple testing by computing FDR q-values using the Benjamini-Hochberg procedure (38). For GSSG, differences between groups were tested by two-tailed Mann-Whitney U test. The relation between the DNA methylation level of TF binding regions and mRNA expression was described using the Pearson correlation coefficient with log transformed mRNA expression levels. *P* < 0.05 or q < 0.05 was considered significant. All statistical analyses were performed in R (R Foundation for Statistical Computing, Vienna, Austria).

## RESULTS

### A prenatal HF diet alters hepatic expression of gene sets involved in lipid metabolism, oxidative stress response, and cell proliferation

Previous work showed that, at 12 weeks of age, HF/HF offspring displayed postprandial hypertriglyceridemia and an impaired clearance of lipids from the blood into the liver after a HF meal (24). To study whether this impaired metabolic phenotype in HF/HF offspring was related to alterations in hepatic gene expression, a microarray was performed on offspring livers at 12 and 28 weeks of age. As a result, a total of 492 genes were found to be differentially expressed in 12-week-old HF/HF offspring, compared with LF/HF offspring. Moreover, GSEA revealed that the prenatal HF diet significantly affected expression of four different pathways in offspring livers at 12 weeks of age, with two being upregulated and two downregulated (**Table 1**). The two downregulated pathways were related to hepatic lipid biosynthesis and uptake (i.e., *Activation of gene expression by SREBP*) and cellular movement (i.e., *Striated muscle contraction*), whereas the upregulated pathways were linked to oxidative stress response [i.e., *nuclear factor (erythroid-derived 2)-like 2* (*Nrf2*) *targets*] and intracellular transport (i.e., *Kinesins*).

**TABLE 1.** Pathways significantly affected by prenatal HF diet in offspring livers at 12 weeks (n = 7 per group) and 28 weeks (n = 9 per group) of age

Biological Function of Pathway	Pathway	NES	FDR
12 weeks			
Cellular movement	Striated muscle contraction*^a^*	−2.34	0.004
Lipid biosynthesis/uptake	Activation of gene expression by SREBP*^b^*	−2.32	0.002
Oxidative stress response	Nrf2 targets*^b^*	2.60	<0.000
Intracellular transport	Kinesins*^b^*	2.08	0.015
28 weeks			
Lipid biosynthesis/uptake	Cholesterol biosynthesis*^b^*	−2.29	0.002
	Activation of gene expression by SREBP*^b^*	−1.95	0.006
	Steroid biosynthesis*^c^*	−1.91	0.009
Mitochondrial translation	Mitochondrial translation initiation*^b^*	−2.28	0.001
	Mitochondrial translation elongation*^b^*	−2.28	0.001
	Mitochondrial translation termination*^b^*	−2.20	0.001
Antigen processing/presentation	Cross-presentation of soluble exogenous antigens (endosomes)*^b^*	−2.22	0.001
	Antigen processing-cross presentation*^b^*	−1.77	0.028
	ER-phagosome pathway*^b^*	−1.84	0.016
Mitosis/cell cycle	Autodegradation of Cdh1 by Cdh1:APC/C*^b^*	−2.20	0.001
	APC/C:Cdc20-mediated degradation of mitotic proteins*^b^*	−2.18	0.001
	APC/C:Cdc20-mediated degradation of Securin*^b^*	−2.18	0.001
Proteasomal degradation	Proteasome*^c^*	−2.15	0.001
	Proteasome pathway*^d^*	−2.11	0.001
	Proteasome degradation*^a^*	−1.99	0.005
HIV-host interaction	Vpu mediated degradation of CD4*^b^*	−1.93	0.008
	Vif-mediated degradation of APOBEC3G*^b^*	−1.93	0.008
Regulation of transcription and translation	Ribosome biogenesis in eukaryotes*^c^*	−2.00	0.005
	Eukaryotic transcription initiation*^a^*	−1.91	0.009
	RNA transport*^c^*	−1.89	0.011
Wnt signaling	Degradation of axin*^b^*	−1.89	0.011
	Asymmetric localization of PCP proteins*^b^*	−1.88	0.011
	Degradation of DVL*^b^*	−1.80	0.022
Hedgehog signaling	Hedgehog ligand biogenesis disease*^b^*	−1.80	0.022
	Degradation of GLI1 by the proteasome*^b^*	−1.79	0.023
	Processing defective Hh variants are degraded by the proteasome*^b^*	−1.78	0.027
Sema4D signaling	Sema4D in semaphorin signaling*^b^*	2.11	0.040
	Sema4D-induced cell migration and growth cone collapse*^b^*	2.06	0.038

For each biological function, the top three (based on NES) significant pathways are shown. A negative or positive NES indicates that the pathway was downregulated or upregulated, respectively, in HF/HF offspring compared with LF/HF offspring. A complete overview of all significantly affected pathways is listed in supplemental Table S3.

a WikiPathways database.

b Reactome database.

c KEGG database.

d BioCarta database.

In 28-week-old offspring, expression of 534 individual genes was altered in HF/HF offspring compared with LF/HF offspring. Furthermore, the prenatal HF diet significantly affected expression of 63 pathways of which two pathways were upregulated and 61 pathways were downregulated. The two upregulated pathways were both related to Sema4D signaling. Of the downregulated pathways, however, the majority were involved in cell proliferation, having functions in mitosis, cell cycle, proteasomal degradation, Wnt signaling, and Hedgehog signaling. In addition, hepatic lipid biosynthesis and uptake was still downregulated in 28-week-old HF/HF offspring, including gene sets *Activation of gene expression by SREBP*, *Cholesterol biosynthesis*, and *Steroid biosynthesis*.

Altogether, a prenatal HF diet altered hepatic expression of gene sets involved in lipid biosynthesis and uptake, oxidative stress response. and cell proliferation.

### A prenatal HF diet increases oxidative stress in offspring livers

**Fig. 3.**
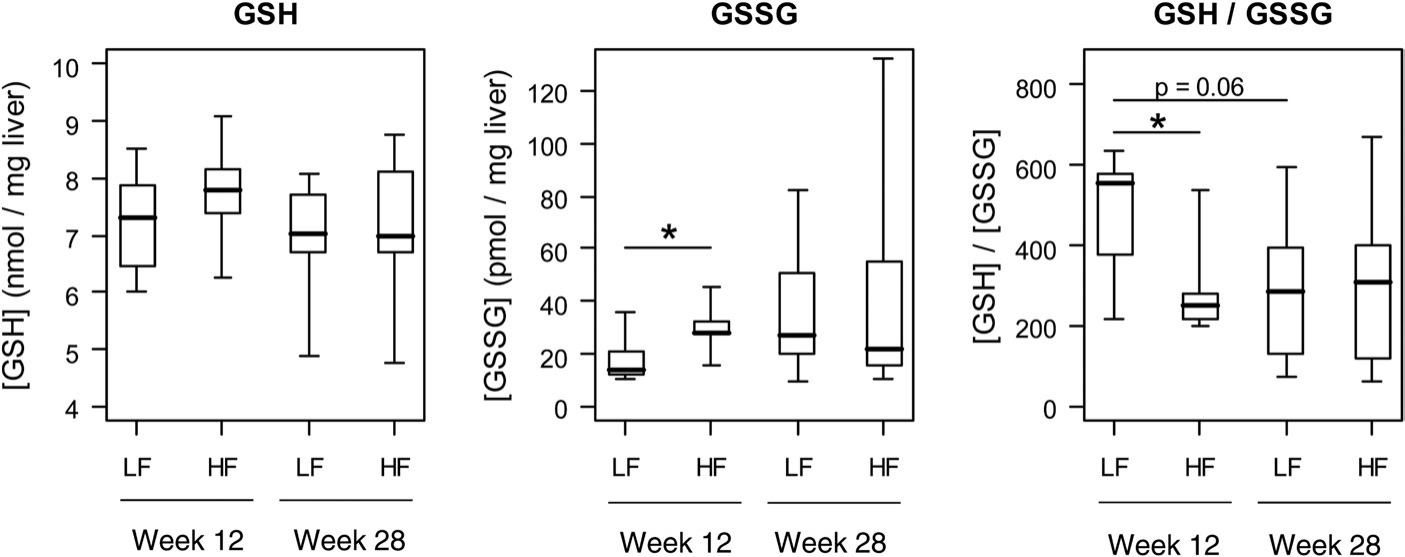
GSH and GSSG concentrations and the ratio between GSH and GSSG concentrations in 12-week-old (n = 7 for LF and n = 6 for HF) and 28-week-old (n = 9 per group) offspring livers after both parent mice received either a prenatal LF or HF diet. Boxplots represent minimum, first quartile, median, second quartile, and maximum. *P < 0.05, based on two-tailed independent samples *t*-test (GSH and GSH/GSSG) or two-tailed Mann-Whitney U test (GSSG).

Because the prenatal HF diet upregulated hepatic expression of the Nrf2 pathway in 12-week-old offspring, we next examined whether livers of these mice also presented increased levels of oxidative stress, which was done by measuring GSH and GSSG. The results show that GSH levels were similar in all four groups, whereas GSSG levels were significantly increased in HF/HF offspring compared with LF/HF at 12 weeks of age (**Fig. 3**). Consequently, the GSH/GSSG ratio was significantly decreased in HF/HF offspring compared with LF/HF at 12 weeks of age, indicating an increased exposure to oxidative stress in the livers of HF/HF mice. In addition, there was a tendency (*P* = 0.06) for decreased GSH/GSSG ratio in 28-week-old compared with 12-week-old LF/HF offspring, indicating that oxidative stress tended to increase over time in the control group.

### A prenatal HF diet induces hypermethylation of TF binding sites upstream of *Lpin1*

**Fig. 4.**
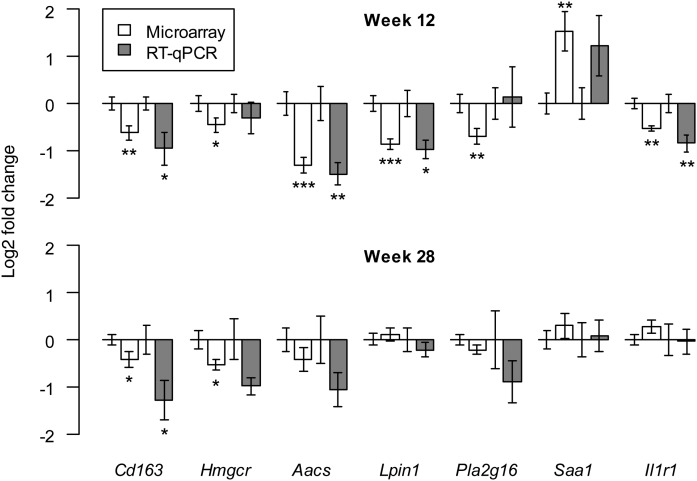
Effect of prenatal HF diet on hepatic mRNA expression of *Cd163*, *Hmgcr*, *Aacs*, *Lpin1*, *Pla2g16*, *Saa1*, and *Il1r1* in 12-week-old (n = 7 per group) and 28-week-old offspring (n = 9 per group) analyzed by microarray and real-time qPCR. Expression was normalized against *Actb* and *Gapdh* and presented relative to control (LF/HF) expression (controls have Log2-fold change of 0). Bars indicate mean ± SEM. *P < 0.05; **P < 0.01; ***P < 0.001, based on two-tailed paired intensity-based moderated t-statistics (microarray) or two-tailed independent samples *t*-test (real-time qPCR).

To examine whether the observed effect of a prenatal HF diet on hepatic gene expression is regulated by alterations in DNA methylation, further analysis was performed on the candidate genes, *Cd163*, *Hmgcr*, *Aacs*, *Lpin1*, *Pla2g16*, *Saa1*, and *Il1r1*. These genes were selected based on their involvement in lipid metabolism (*Hmgcr*, *Aacs*, *Lpin1*, and *Pla2g16*) or inflammation (*Cd163*, *Saa1*, and *Il1r1*) and their significant differential expression in the microarray. This differential expression was verified by real-time qPCR, which showed that the prenatal HF diet significantly altered hepatic mRNA expression of *Cd163* and *Hmgcr* at 12 and 28 weeks of age, whereas expression of *Aacs*, *Lpin1*, *Saa1*, and *Il1r1* was only significantly changed at week 12 (**Fig. 4**). For *Pla2g16*, differential expression could not be confirmed with real-time qPCR, and it was therefore not included for further analyses.

**Fig. 5.**
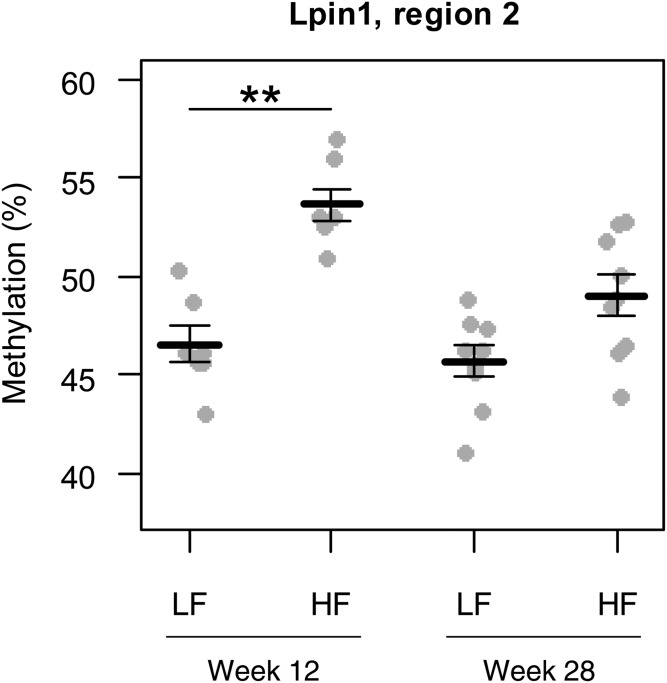
Effect of prenatal HF diet on offspring hepatic DNA methylation of *Lpin1* TF binding region 2 (Chr12: 16,590,890–16,591,130 bp) at 12 weeks (n = 7 per group) and 28 weeks of age (n = 9 per group). Lines and error bars indicate mean ± SEM. **FDR q-value <0.01, based on two-tailed independent samples *t*-test followed by correction for multiple testing using the Benjamini-Hochberg procedure.

For each of the confirmed genes, methylation was measured at CpGs within TF binding sites, thereby having the potential to regulate expression of that gene. Analysis showed that, at 12 weeks of age, hepatic DNA methylation of *Lpin1* TF binding region 2 (Chr12: 16,590,890–16,591,130 bp) was significantly increased (Δ = 7.1%, q = 0.002) in HF/HF offspring compared with LF/HF offspring (**Fig. 5**). At 28 weeks of age, there was no longer a significant difference, with *Lpin1* region 2 DNA methylation levels of HF/HF offspring partly being restored to the level of LF/HF offspring. No significant changes were observed for *Lpin1* TF binding regions 1 and 3 or for any TF binding region upstream of *Cd163*, *Hmgcr*, *Aacs*, *Saa1*, and *Il1r1* at 12 or 28 weeks of age (supplemental Fig. S1).

### mRNA expression of *Lpin1* correlates with DNA methylation of *Lpin1* TF binding sites

The observed hypermethylation of *Lpin1* TF binding region 2 in 12-week-old HF/HF offspring was concurrent with a decrease in mRNA expression of *Lpin1*, indicating a negative relation between DNA methylation and gene expression for this locus. Existence of a correlation, however, would provide a stronger indication for DNA methylation to be involved in regulation of gene expression. Therefore, Pearson correlation was assessed for mean DNA methylation levels of TF binding regions versus microarray expression levels of the corresponding gene. This analysis showed that, at 12 weeks of age, *Lpin1* mRNA expression correlated significantly with DNA methylation of *Lpin1* TF binding region 2 (*r* = −0.54, *P* < 0.05). Furthermore, *Saa1* mRNA expression and DNA methylation of *Saa1* TF binding region 1 also correlated significantly (*r* = 0.53; *P* < 0.05). At 28 weeks of age, both of these correlations were no longer significant. However, there was a significant correlation between *Lpin1* mRNA expression and DNA methylation of *Lpin1* TF binding region 3 (*r* = 0.50, *P* < 0.05) at 28 weeks of age. Interestingly, this *Lpin1* region 3 is a reported binding site for both of the TFs, Nrf2 and SREBP-1 (obtained from GTRD, version 17.04).

## DISCUSSION

While the phenotypic effects of a prenatal HF diet on offspring have been studied thoroughly, it is less well-known through which mechanisms these long-lasting effects are transmitted. Therefore, we studied the effect of a combined paternal and maternal HF diet on hepatic DNA methylation and gene expression in adult mouse offspring. We showed that this prenatal HF diet altered hepatic DNA methylation and gene expression of pathways involved in lipid metabolism, oxidative stress response, and cell proliferation. We hypothesize that these changes underlie the previously reported postprandial hypertriglyceridemia and decrease hepatic lipid clearance in offspring exposed to a prenatal HF diet (24).

First, we showed that a prenatal HF diet consistently downregulated the gene set *Activation of gene expression by SREBP* at both 12 and 28 weeks of age. SREBP represents a group of TFs that regulate expression of genes involved in fatty acid and cholesterol synthesis and uptake (39–44). Because SREBP targets are involved in lipid uptake, the observed downregulation of hepatic SREBP signaling in HF/HF offspring may contribute to the impaired postprandial hepatic lipid clearance, as was previously reported in these offspring mice (24). The observed downregulation of SREBP signaling is in agreement with two previous studies that examined the effect of a maternal HF diet on offspring livers. These studies reported that SREBP gene and protein expression were diminished and that this regulated the observed decrease in mRNA expression of genes involved in fatty acid, cholesterol, and steroid biosynthesis, and other SREBP-related functions (45, 46). In contrast, two other studies found an increase in mRNA expression of *Srebp1c* and some of its downstream genes involved in de novo lipogenesis in response to a prenatal HF diet (13, 14). Although the reasons for these opposite effects on SREBP expression in similar experimental settings remain unclear, these reports do underline the involvement of SREBP in the effects of a prenatal HF diet on offspring livers.

Next to a decreased hepatic lipid uptake induced by SREBP downregulation, alterations in the metabolic phenotype of HF/HF offspring can also be a result of an increased export of lipids from the liver into the circulation. Such an increased export may be facilitated by the upregulation of the pathway *Kinesins* in HF/HF offspring. Kinesins are motor proteins that are, among other processes, required for intracellular transport of TG-rich lipid droplets to the smooth ER in hepatocytes (47). This transport enables lipid droplets to be catabolized and TGs to be available for VLDL assembly and subsequent secretion from the liver. Upregulation of these kinesin motors could therefore contribute to enhanced VLDL-mediated export of lipids from the liver to the blood, thereby adding to the previously reported trend for decreased fasted hepatic lipid content in HF/HF offspring (24).

Besides the *Kinesins* pathway, the prenatal HF diet also increased the expression of the gene set *Nrf2 targets* in 12-week-old offspring. Nrf2 is a TF that regulates expression of antioxidant proteins that protect against oxidative damage triggered by injury and inflammation (48–50). To examine whether these mice also displayed increased levels of oxidative stress, intracellular levels of GSSG and GSH were measured in offspring livers. This showed that at 12 weeks of age, oxidative stress levels were indeed significantly increased in HF/HF offspring livers compared with LF/HF. At 28 weeks of age, however, GSSG and GSH levels, as well as expression of the Nrf2 pathway, were no longer different between HF/HF and LF/HF offspring. Instead, both groups displayed elevated levels of oxidative stress, similar to the levels observed in 12-week-old HF/HF offspring. These findings suggest that the postweaning HF diet led to a gradual increase in oxidative stress levels in livers, irrespective of the parents’ diet. If the parents were additionally exposed to a HF diet, the increased levels of oxidative stress were already reached at 12 weeks of age, thereby prolonging the time that offspring livers were exposed to oxidative stress. This link between a HF diet and oxidative stress response has been indicated before by studies showing that pharmacologic or transgenic activation of Nrf2 protects mice from the effects of a HF diet, such as obesity, insulin resistance, and liver steatosis (51, 52). Furthermore, it has previously been reported that exposure to a prenatal HF diet affected not only hepatic mRNA levels (17, 53) but also DNA methylation of Nrf2-related genes (17), implying that epigenetics may regulate the effects of a prenatal HF diet on oxidative stress response.

To further investigate the involvement of epigenetics in the effects of a prenatal HF diet, DNA methylation was measured at CpGs within TF binding sites of selected differentially expressed genes. We found that the prenatal HF diet hypermethylated a TF binding region upstream of *Lpin1*. In addition, DNA methylation of two *Lpin1* TF binding regions correlated significantly with *Lpin1* gene expression. The observed correlations suggest that, in the current study, *Lpin1* mRNA expression was (at least partly) regulated by DNA methylation. The Lpin1 protein has several biological functions, depending on the tissue in which it is expressed (54–56). In liver, Lpin1 mainly functions as a transcriptional coactivator and its knockdown leads to increased VLDL secretion and elevated plasma TGs (55). Therefore, hypermethylation of *Lpin1* TF binding regions and subsequent downregulation of *Lpin1* expression may explain the phenotypic changes in HF/HF offspring, including increased circulatory TGs and decreased hepatic lipids.

In addition to *Lpin1*, a correlation was also observed between a TF binding region and mRNA expression for *Saa1*. *Saa1* is an acute phase protein mainly produced by hepatocytes in response to inflammation and tissue damage. Normally, the liver is able to recover from tissue damage (57–59). In the current study, however, pathway analysis of microarray data showed that the prenatal HF diet downregulated expression of various gene sets involved in mitosis, cell cycle, proteasomal degradation, Wnt signaling, and Hedgehog signaling in offspring livers. Although we do not have phenotypical data to further support this, these results are an indication that cell proliferation is reduced in HF/HF offspring, possibly leading to impaired recovery from liver damage. These findings are in line with a previous study, which reported that rat offspring of mothers fed a HF diet showed hepatic cell cycle inhibition, together with related changes in gene expression and DNA methylation (60). Similarly, many genes involved in cell growth, differentiation, proliferation, and development were both differentially expressed and differentially methylated in the livers of offspring of HF diet-fed mothers (17). Furthermore, it has been shown that exposure to HF diet not only before birth but also after birth results in decreased liver proliferation, indicated by impaired liver regeneration after partial hepatectomy in mice (61, 62). Therefore, alterations in tissue damage and cell proliferation in the liver after a prenatal HF diet deserve further attention in future research.

In comparison to previous reports, our study has a few advantages. First, whereas most studies only examined the effects of a prenatal HF diet at one specific offspring age, we performed measurements in offspring at 12 and 28 weeks of age. This enabled us to analyze alterations in gene expression and DNA methylation over time. Second, many studies only focus either on the phenotypic outcome of a prenatal HF diet or on mechanisms underlying these effects. In our experiment, previously published effects on the metabolic phenotype of offspring were now linked to alterations in gene expression, DNA methylation, and oxidative stress. Finally, most studies examine the effects of a prenatal HF diet by combining a maternal HF diet with a paternal LF diet or vice versa. However, in our study, both father and mother were fed the same diet to better mimic the current Western dietary situation in which family members share eating patterns (19). In addition, combining a maternal and paternal HF diet can have additive effects on the offspring’s metabolic health, resulting in more prominent health effects in offspring when compared with separate maternal or paternal HF diets (14, 16).

With the current study design, we did not assess the effect of a postweaning LF diet. All offspring was weaned onto a HF diet, because this experimental setup provides a better model for the current Western type of diet, which is characterized by a HF content. Furthermore, it has been shown that a postweaning HF diet may aggravate the negative effects of the prenatal HF diet (15). Another study limitation is that the analyses were limited to male offspring. Sex differences occur in response to a prenatal HF diet, with male offspring having a larger increase in bodyweight and plasma TG (23), whereas female offspring have higher blood pressure and leptin levels (20, 21). Likewise, exposure to a prenatal HF diet affects hepatic gene expression differently in male and female offspring (23). Major contributors to these sex dimorphisms are estrogens and androgens (63, 64). In females, these effects depend on the hormonal cycle, making female mice more prone to variation in metabolic outcomes. Therefore, only male offspring were included in present study.

In summary, we showed that a combined maternal and paternal HF diet alters hepatic gene expression of pathways related to lipid synthesis and uptake, oxidative stress response, and cell proliferation. The downregulation of lipid uptake could account for the previously observed decreased postprandial hepatic lipid uptake and hypertriglyceridemia in HF/HF offspring. Furthermore, the upregulation of the Nrf2-mediated oxidative stress response was confirmed by assessment of GSH/GSSG ratios in the livers of offspring. In other words, the changes in gene expression due to prenatal HF diet were mechanistically linked to phenotypic alterations. We propose that the observed effects on the offspring’s metabolic phenotype and gene expression are driven by alterations in DNA methylation, as indicated by the hypermethylation of TF binding sites upstream of *Lpin1* and the correlation between DNA methylation of *Lpin1* TF binding sites and gene expression of *Lpin1*. Society may have to invest in early life nutrition to prevent common diseases occurring in offspring at older ages. More knowledge is needed to unravel the molecular mechanisms, including DNA methylation and other epigenetic mechanisms that underlie the negative effects of a prenatal HF diet on offspring health.

## Supplementary Material

Supplemental Data

## References

[1] Barker D. J. 1997 Maternal nutrition, fetal nutrition, and disease in later life. Nutrition. 13: 807–813. 9290095 10.1016/s0899-9007(97)00193-7

[2] de Rooij S. R., Painter R. C., Roseboom T. J., Phillips D. I., Osmond C., Barker D. J., Tanck M. W., Michels R. P., Bossuyt P. M., and Bleker O. P. 2006 Glucose tolerance at age 58 and the decline of glucose tolerance in comparison with age 50 in people prenatally exposed to the Dutch famine. Diabetologia. 49: 637–643. 16470406 10.1007/s00125-005-0136-9

[3] Ravelli A. C., van der Meulen J. H., Michels R. P., Osmond C., Barker D. J., Hales C. N., and Bleker O. P. 1998 Glucose tolerance in adults after prenatal exposure to famine. Lancet. 351: 173–177. 9449872 10.1016/s0140-6736(97)07244-9

[4] Ravelli A. C. J., van der Meulen J. H. P., Osmond C., Barker D. J. P., and Bleker O. P. 1999 Obesity at the age of 50 y in men and women exposed to famine prenatally. Am. J. Clin. Nutr. 70: 811–816. 10539740 10.1093/ajcn/70.5.811

[5] Roseboom T. J., van der Meulen J. H., Osmond C., Barker D. J., Ravelli A. C., Schroeder-Tanka J. M., van Montfrans G. A., Michels R. P., and Bleker O. P. 2000 Coronary heart disease after prenatal exposure to the Dutch famine, 1944–45. Heart. 84: 595–598. 11083734 10.1136/heart.84.6.595 PMC1729504

[6] Cooper R., Hyppönen E., Berry D., and Power C. 2010 Associations between parental and offspring adiposity up to midlife: the contribution of adult lifestyle factors in the 1958 British Birth Cohort Study. Am. J. Clin. Nutr. 92: 946–953. 20702606 10.3945/ajcn.2010.29477

[7] Li L., Law C., Lo Conte R., and Power C. 2009 Intergenerational influences on childhood body mass index: the effect of parental body mass index trajectories. Am. J. Clin. Nutr. 89: 551–557. 19106237 10.3945/ajcn.2008.26759

[8] Eriksson J. G., Sandboge S., Salonen M. K., Kajantie E., and Osmond C. 2014 Long-term consequences of maternal overweight in pregnancy on offspring later health: findings from the Helsinki Birth Cohort Study. Ann. Med. 46: 434–438. 24910160 10.3109/07853890.2014.919728

[9] Gaillard R., Steegers E. A., Duijts L., Felix J. F., Hofman A., Franco O. H., and Jaddoe V. W. 2014 Childhood cardiometabolic outcomes of maternal obesity during pregnancy: the Generation R Study. Hypertension. 63: 683–691. 24379180 10.1161/HYPERTENSIONAHA.113.02671

[10] Hochner H., Friedlander Y., Calderon-Margalit R., Meiner V., Sagy Y., Avgil-Tsadok M., Burger A., Savitsky B., Siscovick D. S., and Manor O. 2012 Associations of maternal prepregnancy body mass index and gestational weight gain with adult offspring cardiometabolic risk factors: the Jerusalem Perinatal Family Follow-up Study. Circulation. 125: 1381–1389. 22344037 10.1161/CIRCULATIONAHA.111.070060 PMC3332052

[11] Yu Z., Han S., Zhu J., Sun X., Ji C., and Guo X. 2013 Pre-pregnancy body mass index in relation to infant birth weight and offspring overweight/obesity: a systematic review and meta-analysis. PLoS One. 8: e61627. 23613888 10.1371/journal.pone.0061627 PMC3628788

[12] Keleher M. R., Zaidi R., Shah S., Oakley M. E., Pavlatos C., El Idrissi S., Xing X., Li D., Wang T., and Cheverud J. M. 2018 Maternal high-fat diet associated with altered gene expression, DNA methylation, and obesity risk in mouse offspring. PLoS One. 13: e0192606. 29447215 10.1371/journal.pone.0192606 PMC5813940

[13] Pruis M. G., Lendvai A., Bloks V. W., Zwier M. V., Baller J. F., de Bruin A., Groen A. K., and Plösch T. 2014 Maternal western diet primes non–alcoholic fatty liver disease in adult mouse offspring. Acta Physiol. (Oxf.). 210: 215–227. 24224789 10.1111/apha.12197

[14] Ornellas F., Souza-Mello V., Mandarim-de-Lacerda C. A., and Aguila M. B. 2015 Programming of obesity and comorbidities in the progeny: lessons from a model of diet-induced obese parents. PLoS One. 10: e0124737. 25880318 10.1371/journal.pone.0124737 PMC4399989

[15] Fullston T., McPherson N. O., Owens J. A., Kang W. X., Sandeman L. Y., and Lane M. 2015 Paternal obesity induces metabolic and sperm disturbances in male offspring that are exacerbated by their exposure to an “obesogenic” diet. Physiol. Rep. 3: e12336. 25804263 10.14814/phy2.12336 PMC4393169

[16] Masuyama H., Mitsui T., Eguchi T., Tamada S., and Hiramatsu Y. 2016 The effects of paternal high-fat diet exposure on offspring metabolism with epigenetic changes in the mouse adiponectin and leptin gene promoters. Am. J. Physiol. Endocrinol. Metab. 311: E236–E245. 27245335 10.1152/ajpendo.00095.2016

[17] Seki Y., Suzuki M., Guo X., Glenn A. S., Vuguin P. M., Fiallo A., Du Q., Ko Y-A., Yu Y., and Susztak K. 2017 In utero exposure to a high-fat diet programs hepatic hypermethylation and gene dysregulation and development of metabolic syndrome in male mice. Endocrinology. 158: 2860–2872. 28911167 10.1210/en.2017-00334 PMC5659663

[18] Soubry A., Murphy S., Wang F., Huang Z., Vidal A., Fuemmeler B., Kurtzberg J., Murtha A., Jirtle R., and Schildkraut J. 2015 Newborns of obese parents have altered DNA methylation patterns at imprinted genes. Int. J. Obes. (Lond.). 39: 650–657. 24158121 10.1038/ijo.2013.193 PMC4048324

[19] Berge J. M., Wall M., Larson N., Forsyth A., Bauer K. W., and Neumark-Sztainer D. 2014 Youth dietary intake and weight status: healthful neighborhood food environments enhance the protective role of supportive family home environments. Health Place. 26: 69–77. 24378461 10.1016/j.healthplace.2013.11.007 PMC3942084

[20] Khan I., Dekou V., Hanson M., Poston L., and Taylor P. 2004 Predictive adaptive responses to maternal high-fat diet prevent endothelial dysfunction but not hypertension in adult rat offspring. Circulation. 110: 1097–1102. 15326063 10.1161/01.CIR.0000139843.05436.A0

[21] Bellisario V., Berry A., Capoccia S., Raggi C., Panetta P., Branchi I., Piccaro G., Giorgio M., Pelicci P. G., and Cirulli F. 2014 Gender-dependent resiliency to stressful and metabolic challenges following prenatal exposure to high-fat diet in the p66Shc-/- mouse. Front. Behav. Neurosci. 8: 285. 25202246 10.3389/fnbeh.2014.00285 PMC4141279

[22] Miller C., Krishna S., Lin Z., Della-Fera M. A., Harn D., de la Serre C., Baile C., and Filipov N. 2014 Early sex differences in hepatic metabolic signaling in offspring of obese female mice (Abstract). FASEB J. 28 (Suppl. 1): 1033.11.

[23] Mischke M., Pruis M. G., Boekschoten M. V., Groen A. K., Fitri A. R., van de Heijning B. J., Verkade H. J., Müller M., Plösch T., and Steegenga W. T. 2013 Maternal Western-style high fat diet induces sex-specific physiological and molecular changes in two-week-old mouse offspring. PLoS One. 8: e78623. 24223833 10.1371/journal.pone.0078623 PMC3818485

[24] van Ewijk P. A., Paglialunga S., Kooi M. E., Nunes P. M., Gemmink A., Slenter J., Kornips E., Jorgensen J. A., Hoeks J., Wildberger J. E., 2015 Effects of high-fat feeding on ectopic fat storage and postprandial lipid metabolism in mouse offspring. Obesity (Silver Spring). 23: 2242–2250. 26530934 10.1002/oby.21235

[25] Lin K., Kools H., de Groot P. J., Gavai A. K., Basnet R. K., Cheng F., Wu J., Wang X., Lommen A., and Hooiveld G. J. 2011 MADMAX - management and analysis database for multiple ∼omics experiments. J. Integr. Bioinform. 8: 160. 21778530 10.2390/biecoll-jib-2011-160

[26] Bolstad B. M., Irizarry R. A., Åstrand M., and Speed T. P. 2003 A comparison of normalization methods for high density oligonucleotide array data based on variance and bias. Bioinformatics. 19: 185–193. 12538238 10.1093/bioinformatics/19.2.185

[27] Subramanian A., Tamayo P., Mootha V. K., Mukherjee S., Ebert B. L., Gillette M. A., Paulovich A., Pomeroy S. L., Golub T. R., and Lander E. S. 2005 Gene set enrichment analysis: a knowledge-based approach for interpreting genome-wide expression profiles. Proc. Natl. Acad. Sci. USA. 102: 15545–15550. 16199517 10.1073/pnas.0506580102 PMC1239896

[28] Nishimura D. 2001 BioCarta. Biotech. Software & Internet Report. 2: 117–120.

[29] Kanehisa M., and Goto S. 2000 KEGG: Kyoto encyclopedia of genes and genomes. Nucleic Acids Res. 28: 27–30. 10592173 10.1093/nar/28.1.27 PMC102409

[30] Fabregat A., Sidiropoulos K., Garapati P., Gillespie M., Hausmann K., Haw R., Jassal B., Jupe S., Korninger F., and McKay S. 2016 The reactome pathway knowledgebase. Nucleic Acids Res. 44 (D1): D481–D487. 26656494 10.1093/nar/gkv1351 PMC4702931

[31] Slenter D. N., Kutmon M., Hanspers K., Riutta A., Windsor J., Nunes N., Mélius J., Cirillo E., Coort S. L., and Digles D. 2018 WikiPathways: a multifaceted pathway database bridging metabolomics to other omics research. Nucleic Acids Res. 46 (D1): D661–D667. 29136241 10.1093/nar/gkx1064 PMC5753270

[32] Sartor M. A., Tomlinson C. R., Wesselkamper S. C., Sivaganesan S., Leikauf G. D., and Medvedovic M. 2006 Intensity-based hierarchical Bayes method improves testing for differentially expressed genes in microarray experiments. BMC Bioinformatics. 7: 538. 17177995 10.1186/1471-2105-7-538 PMC1781470

[33] Wang X., and Seed B. 2003 A PCR primer bank for quantitative gene expression analysis. Nucleic Acids Res. 31: e154. 14654707 10.1093/nar/gng154 PMC291882

[34] Cui W., Taub D. D., and Gardner K. 2007 qPrimerDepot: a primer database for quantitative real time PCR. Nucleic Acids Res. 35: D805–D809. 17068075 10.1093/nar/gkl767 PMC1635330

[35] Livak K. J., and Schmittgen T. D. 2001 Analysis of relative gene expression data using real-time quantitative PCR and the 2(-Delta Delta C(T)) method. Methods. 25: 402–408. 11846609 10.1006/meth.2001.1262

[36] Yevshin I., Sharipov R., Valeev T., Kel A., and Kolpakov F. 2017 GTRD: a database of transcription factor binding sites identified by ChIP-seq experiments. Nucleic Acids Res. 45 (D1): D61–D67. 27924024 10.1093/nar/gkw951 PMC5210645

[37] Rahman I., Kode A., and Biswas S. K. 2006 Assay for quantitative determination of glutathione and glutathione disulfide levels using enzymatic recycling method. Nat. Protoc. 1: 3159–3165. 17406579 10.1038/nprot.2006.378

[38] Benjamini Y., and Hochberg Y. 1995 Controlling the false discovery rate: a practical and powerful approach to multiple testing. J. R. Stat. Soc. B Stat Methodol. 57: 289–300.

[39] Horton J. D., Goldstein J. L., and Brown M. S. 2002 SREBPs: activators of the complete program of cholesterol and fatty acid synthesis in the liver. J. Clin. Invest. 109: 1125–1131. 11994399 10.1172/JCI15593 PMC150968

[40] Sakakura Y., Shimano H., Sone H., Takahashi A., Inoue K., Toyoshima H., Suzuki S., and Yamada N. 2001 Sterol regulatory element-binding proteins induce an entire pathway of cholesterol synthesis. Biochem. Biophys. Res. Commun. 286: 176–183. [Erratum. 2001. *Biochem. Biophys. Res. Commun.* 287: 311.] 11485325 10.1006/bbrc.2001.5375

[41] Shimano H., Horton J. D., Hammer R. E., Shimomura I., Brown M. S., and Goldstein J. L. 1996 Overproduction of cholesterol and fatty acids causes massive liver enlargement in transgenic mice expressing truncated SREBP-1a. J. Clin. Invest. 98: 1575–1584. 8833906 10.1172/JCI118951 PMC507590

[42] Shimano H., Horton J. D., Shimomura I., Hammer R. E., Brown M. S., and Goldstein J. L. 1997 Isoform 1c of sterol regulatory element binding protein is less active than isoform 1a in livers of transgenic mice and in cultured cells. J. Clin. Invest. 99: 846–854. 9062341 10.1172/JCI119248 PMC507891

[43] Shimano H., Shimomura I., Hammer R. E., Herz J., Goldstein J. L., Brown M. S., and Horton J. D. 1997 Elevated levels of SREBP-2 and cholesterol synthesis in livers of mice homozygous for a targeted disruption of the SREBP-1 gene. J. Clin. Invest. 100: 2115–2124. 9329978 10.1172/JCI119746 PMC508404

[44] Horton J. D., Shimomura I., Brown M. S., Hammer R. E., Goldstein J. L., and Shimano H. 1998 Activation of cholesterol synthesis in preference to fatty acid synthesis in liver and adipose tissue of transgenic mice overproducing sterol regulatory element-binding protein-2. J. Clin. Invest. 101: 2331–2339. 9616204 10.1172/JCI2961 PMC508822

[45] Yu H. L., Miao H. T., Gao L. F., Li L., Xi Y. D., Nie S. P., and Xiao R. 2013 Adaptive responses by mouse fetus to a maternal HLE diet by downregulating SREBP1: a microarray- and bio-analytic-based study. J. Lipid Res. 54: 3269–3280. 23981283 10.1194/jlr.M037416 PMC3826675

[46] Cannon M. V., Buchner D. A., Hester J., Miller H., Sehayek E., Nadeau J. H., and Serre D. 2014 Maternal nutrition induces pervasive gene expression changes but no detectable DNA methylation differences in the liver of adult offspring. PLoS One. 9: e90335. 24594983 10.1371/journal.pone.0090335 PMC3940881

[47] Rai P., Kumar M., Sharma G., Barak P., Das S., Kamat S. S., and Mallik R. 2017 Kinesin-dependent mechanism for controlling triglyceride secretion from the liver. Proc. Natl. Acad. Sci. USA. 114: 12958–12963. 29158401 10.1073/pnas.1713292114 PMC5724275

[48] Yamamoto M., Kensler T. W., and Motohashi H. 2018 The KEAP1-NRF2 system: a thiol-based sensor-effector apparatus for maintaining redox homeostasis. Physiol. Rev. 98: 1169–1203. 29717933 10.1152/physrev.00023.2017 PMC9762786

[49] Itoh K., Chiba T., Takahashi S., Ishii T., Igarashi K., Katoh Y., Oyake T., Hayashi N., Satoh K., and Hatayama I. 1997 An Nrf2/small Maf heterodimer mediates the induction of phase II detoxifying enzyme genes through antioxidant response elements. Biochem. Biophys. Res. Commun. 236: 313–322. 9240432 10.1006/bbrc.1997.6943

[50] Itoh K., Wakabayashi N., Katoh Y., Ishii T., Igarashi K., Engel J. D., and Yamamoto M. 1999 Keap1 represses nuclear activation of antioxidant responsive elements by Nrf2 through binding to the amino-terminal Neh2 domain. Genes Dev. 13: 76–86. 9887101 10.1101/gad.13.1.76 PMC316370

[51] Shin S., Wakabayashi J., Yates M. S., Wakabayashi N., Dolan P. M., Aja S., Liby K. T., Sporn M. B., Yamamoto M., and Kensler T. W. 2009 Role of Nrf2 in prevention of high-fat diet-induced obesity by synthetic triterpenoid CDDO-imidazolide. Eur. J. Pharmacol. 620: 138–144. 19698707 10.1016/j.ejphar.2009.08.022 PMC2752754

[52] Slocum S. L., Skoko J. J., Wakabayashi N., Aja S., Yamamoto M., Kensler T. W., and Chartoumpekis D. V. 2016 Keap1/Nrf2 pathway activation leads to a repressed hepatic gluconeogenic and lipogenic program in mice on a high-fat diet. Arch. Biochem. Biophys. 591: 57–65. 26701603 10.1016/j.abb.2015.11.040 PMC4747866

[53] Terashima M., Barbour S., Ren J., Yu W., Han Y., and Muegge K. 2015 Effect of high fat diet on paternal sperm histone distribution and male offspring liver gene expression. Epigenetics. 10: 861–871. 26252449 10.1080/15592294.2015.1075691 PMC4622005

[54] Chen Y., Rui B-B., Tang L-Y., and Hu C-M. 2015 Lipin family proteins-key regulators in lipid metabolism. Ann. Nutr. Metab. 66: 10–18. 10.1159/000368661 25678092

[55] Finck B. N., Gropler M. C., Chen Z., Leone T. C., Croce M. A., Harris T. E., Lawrence J. C., and Kelly D. P. 2006 Lipin 1 is an inducible amplifier of the hepatic PGC-1α/PPARα regulatory pathway. Cell Metab. 4: 199–210. 16950137 10.1016/j.cmet.2006.08.005

[56] Reue K., and Brindley D. N. 2008 Multiple roles for lipins/phosphatidate phosphatase enzymes in lipid metabolism. J. Lipid Res. 49: 2493–2503. 18791037 10.1194/jlr.R800019-JLR200 PMC2582367

[57] Fausto N., Campbell J. S., and Riehle K. J. 2006 Liver regeneration. Hepatology. 43 (2 Suppl. 1): S45–S53. 16447274 10.1002/hep.20969

[58] Michalopoulos G. K. 2007 Liver regeneration. J. Cell. Physiol. 213: 286–300. 17559071 10.1002/jcp.21172 PMC2701258

[59] Taub R. 2004 Liver regeneration: from myth to mechanism. Nat. Rev. Mol. Cell Biol. 5: 836–847. 15459664 10.1038/nrm1489

[60] Dudley K. J., Sloboda D. M., Connor K. L., Beltrand J., and Vickers M. H. 2011 Offspring of mothers fed a high fat diet display hepatic cell cycle inhibition and associated changes in gene expression and DNA methylation. PLoS One. 6: e21662. 21779332 10.1371/journal.pone.0021662 PMC3133558

[61] DeAngelis R. A., Markiewski M. M., Taub R., and Lambris J. D. 2005 A high–fat diet impairs liver regeneration in C57BL/6 mice through overexpression of the NF–κB inhibitor, IκBα. Hepatology. 42: 1148–1157. 16231352 10.1002/hep.20879

[62] Yang S. Q., Lin H. Z., Mandal A. K., Huang J., and Diehl A. M. 2001 Disrupted signaling and inhibited regeneration in obese mice with fatty livers: implications for nonalcoholic fatty liver disease pathophysiology. Hepatology. 34: 694–706. 11584365 10.1053/jhep.2001.28054

[63] Mauvais-Jarvis F., Clegg D. J., and Hevener A. L. 2013 The role of estrogens in control of energy balance and glucose homeostasis. Endocr. Rev. 34: 309–338. 23460719 10.1210/er.2012-1055 PMC3660717

[64] Navarro G., Allard C., Xu W., and Mauvais–Jarvis F. 2015 The role of androgens in metabolism, obesity, and diabetes in males and females. Obesity (Silver Spring). 23: 713–719. 25755205 10.1002/oby.21033 PMC4380643

