# Supplementary material for: Gene expression and DNA methylation as mechanisms of disturbed metabolism in offspring after exposure to a prenatal HF diet
Source: J Lipid Res. 2019 May 7;60(7):1250–9. doi: 10.1194/jlr.M092593 (PMC6602131; doi:10.1194/jlr.M092593)
Supplement: Supplemental Data [file supp_60_7_1250__index.html]

Gene expression and DNA methylation as mechanisms of disturbed metabolism in offspring after exposure to a prenatal high fat diet — Gene expression and DNA methylation as mechanisms of disturbed metabolism in offspring after exposure to a prenatal HF diet — Supplemental Data 

# Gene expression and DNA methylation as mechanisms of disturbed metabolism in offspring after exposure to a prenatal HF diet

## Supplemental Data

- Revised Supplemental Figure S1 (.pdf, 67 KB) - Supplemental Figure S1
- Revised Supplemental Tables S1+S2 (.xlsx, 33 KB) - Supplemental Tables S1 and S2
- Revised Supplemental Table S3 (.pdf, 72 KB) - Revised Supplemental Table S3
