# Supplementary material for: Gene expression and DNA methylation as mechanisms of disturbed metabolism in offspring after exposure to a prenatal HF diet
Source: J Lipid Res. 2019 May 7;60(7):1250–9. doi: 10.1194/jlr.M092593 (PMC6602131; doi:10.1194/jlr.M092593)
Supplement: Supplemental Data [file 10.1194_M092593_jlr.M092593-1.pdf]

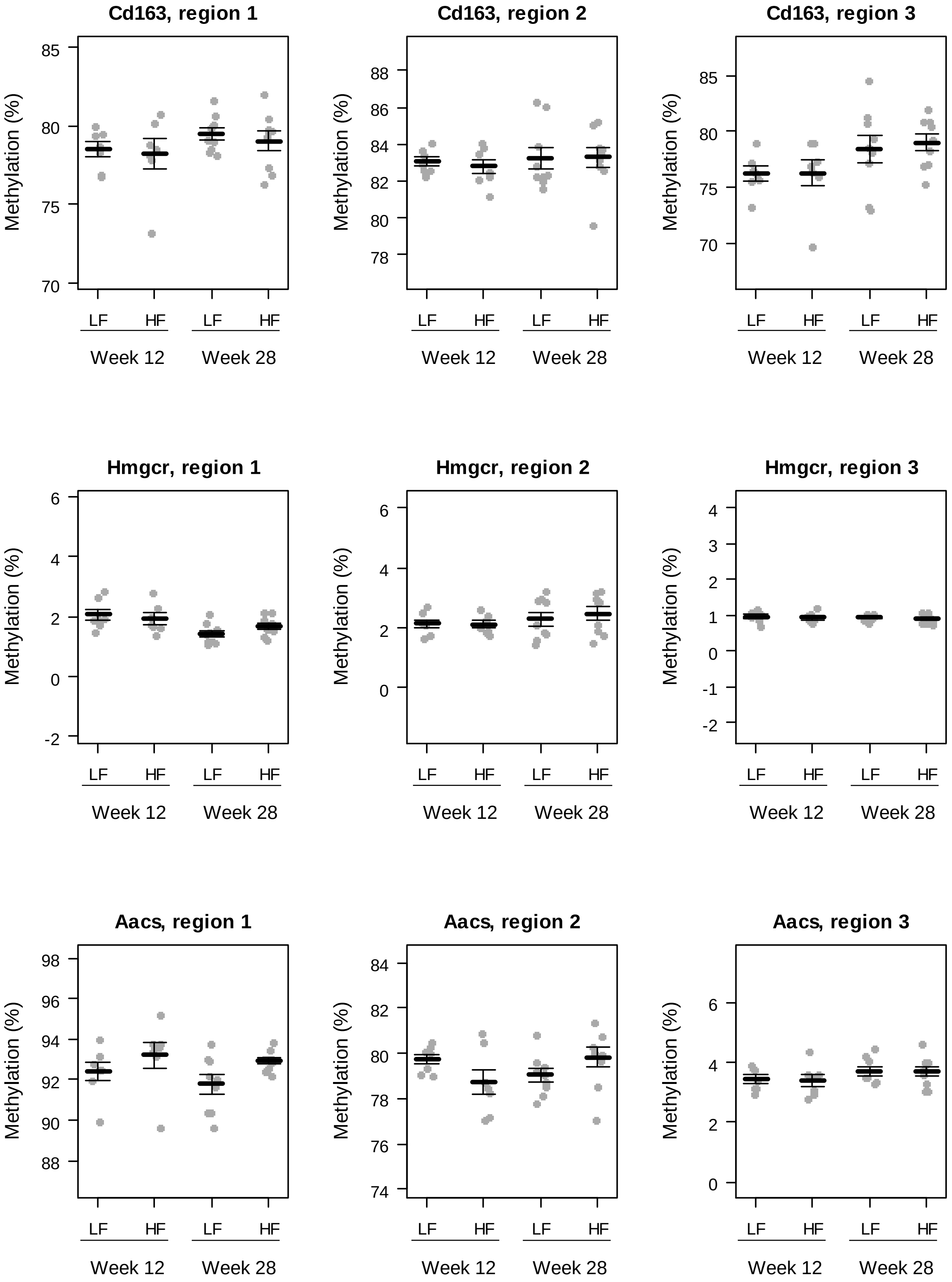
Cd163, region 1
Methylation (%)
70
75
80
85
Cd163, region 2
Methylation (%)
78
80
82
84
86
88
Cd163, region 3
Methylation (%)
70
75
80
85
Hmgcr, region 1
Methylation (%)
-2
0
2
4
6
Hmgcr, region 2
Methylation (%)
0
2
4
6
Hmgcr, region 3
Methylation (%)
-2
-1
0
1
2
3
4
Aacs, region 1
Methylation (%)
88
90
92
94
96
98
Aacs, region 2
Methylation (%)
74
76
78
80
82
84
Aacs, region 3
Methylation (%)
0
2
4
6
LF
HF
LF
HF
Week 12
Week 28

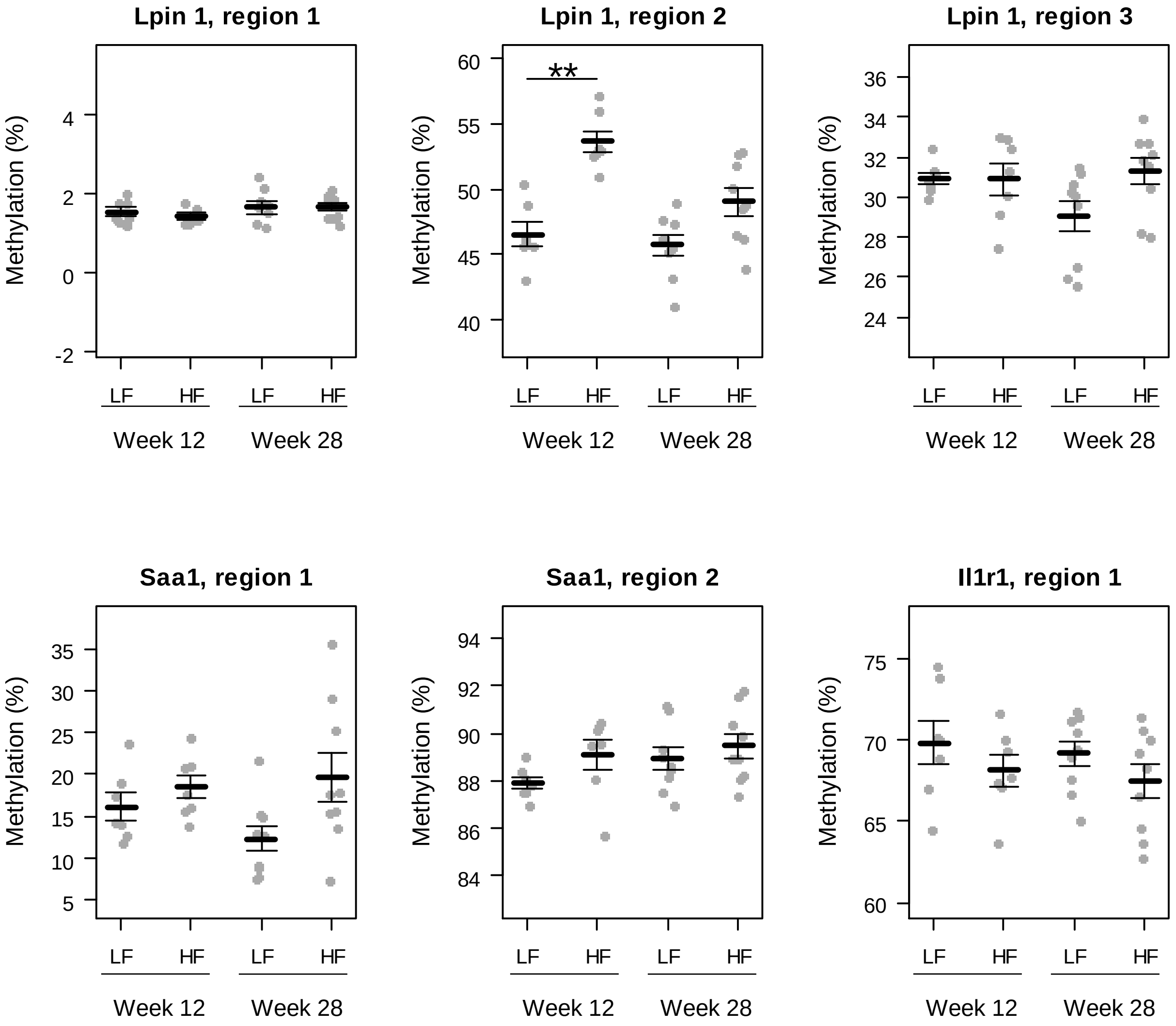

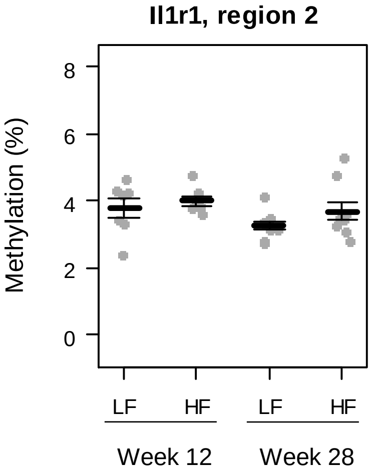

Supplemental Figure S1: Effect of prenatal HF diet on offspring hepatic DNA methylation of TF binding regions upstream of *Cd163*, *Hmgcr*, *Aacs*, *Lpin1*, *Saa1* and *Il1r1* at 12 weeks (n=7 per group) and 28 weeks of age (n=9 per group). Lines and error bars indicate mean ± SEM. **FDR q-value < 0.01.
