# Supplementary material for: Gene expression and DNA methylation as mechanisms of disturbed metabolism in offspring after exposure to a prenatal HF diet
Source: J Lipid Res. 2019 May 7;60(7):1250–9. doi: 10.1194/jlr.M092593 (PMC6602131; doi:10.1194/jlr.M092593)
Supplement: Supplemental Data [file 10.1194_M092593_jlr.M092593-3.pdf]

Supplemental Table S3. A complete overview of all pathways significantly affected by prenatal HF diet in offspring liver at 12 weeks (n=7 per group) and 28 weeks (n=9 per group) of age. A negative or positive NES indicates that the pathway was downregulated or upregulated, respectively, in HF/HF offspring compared to LF/HF offspring.

12 weeks

**Biological function of pathway**	**Pathway**	**NES**	**FDR**
Cellular movement	Striated muscle contraction[a]	-2.34	0.004
Lipid biosynthesis / uptake	Activation of gene expression by SREBP[b]	-2.32	0.002
Oxidative stress response	Nrf2 targets[b]	2.60	<0.000
Intracellular transport	Kinesins[b]	2.08	0.015

28 weeks

**Biological function of pathway**	**Pathway**	**NES**	**FDR**
Lipid biosynthesis / uptake	Cholesterol biosynthesis[b]	-2.29	0.002
	Activation of gene expression by SREBP[b]	-1.95	0.006
	Steroid biosynthesis[c]	-1.91	0.009
Mitochondrial translation	Mitochondrial translation initiation[b]	-2.28	0.001
	Mitochondrial translation elongation[b]	-2.28	0.001
	Mitochondrial translation termination[b]	-2.20	0.001
	Mitochondrial translation[b]	-2.20	0.001
Antigen processing / presentation	Cross-presentation of soluble exogenous antigens (endosomes) [b]	-2.22	0.001
	Antigen processing-Cross presentation[b]	-1.77	0.028
	ER-Phagosome pathway[b]	-1.84	0.016
Mitosis / Cell cycle	Autodegradation of Cdh1 by Cdh1:APC/C[b]	-2.20	0.001
	APC/C:Cdc20 mediated degradation of mitotic proteins[b]	-2.18	0.001
	APC/C:Cdc20 mediated degradation of Securin[b]	-2.18	0.001
	Activation of APC/C and APC/C:Cdc20 mediated degradation of mitotic proteins[b]	-2.16	0.001
	Cdc20:Phospho-APC/C mediated degradation of Cyclin A[b]	-2.14	0.001
	APC/C:Cdh1 mediated degradation of Cdc20 and other APC/C:Cdh1 targeted proteins in late mitosis/early G1[b]	-2.14	0.001

	Stabilization of p53[b]	-2.11	0.001
	Autodegradation of the E3 ubiquitin ligase COP1[b]	-2.10	0.001
	Regulation of activated PAK-2p34 by proteasome mediated degradation[b]	-2.06	0.002
	CDK-mediated phosphorylation and removal of Cdc6[b]	-2.01	0.004
	Regulation of apoptosis[b]	-1.99	0.005
	p53-Independent G1/S DNA damage checkpoint[b]	-1.99	0.005
	Ubiquitin Mediated Degradation of Phosphorylated Cdc25A[b]	-1.98	0.005
	P53 independent DNA damage response[b]	-1.98	0.005
	P53 dependent G1/S DNA damage checkpoint[b]	-1.98	0.005
	SCF(Skp2)-mediated degradation of p27/p21[b]	-1.97	0.005
	Ubiquitin-dependent degradation of Cyclin D1[b]	-1.97	0.005
	P53 dependent G1 DNA damage response[b]	-1.97	0.005
	APC/C-mediated degradation of cell cycle proteins[b]	-1.96	0.006
	Regulation of mitotic cell cycle[b]	-1.95	0.006
	Ubiquitin-dependent degradation of Cyclin D1[b]	-1.95	0.006
	Regulation of APC/C activators between G1/S and early anaphase[b]	-1.94	0.007
	CDT1 association with the CDC6:ORC:origin complex[b]	-1.91	0.009
	Cyclin A:Cdk2-associated events at S phase entry[b]	-1.90	0.009
	G1/S DNA damage checkpoints[b]	-1.90	0.009
	Cyclin E associated events during G1/S transition[b]	-1.84	0.017
	Regulation of ornithine decarboxylase (ODC) [b]	-1.80	0.023
	SCF-beta-TrCP mediated degradation of Emi1[b]	-1.75	0.033
	Assembly of the pre-replicative complex[b]	-1.74	0.034
Proteasomal degradation	Proteasome[c]	-2.15	0.001
	Proteasome pathway[d]	-2.11	0.001
	Proteasome degradation[a]	-1.99	0.005
HIV-host interaction	Vpu mediated degradation of CD4[b]	-1.93	0.008
	Vif-mediated degradation of APOBEC3G[b]	-1.93	0.008
Regulation of transcription and translation	Ribosome biogenesis in eukaryotes[c]	-2.00	0.005
	Eukaryotic transcription initiation[a]	-1.91	0.009
	RNA transport[c]	-1.89	0.011
	AUF1 (hnRNP D0) binds and destabilizes mRNA[b]	-1.85	0.015
	Ribosome[c]	-1.83	0.018
	Basal transcription factors[c]	-1.75	0.033

	Regulation of mRNA stability by proteins that bind AU-rich elements[b]	-1.75	0.033
Wnt signaling	Degradation of axin[b]	-1.89	0.011
	Asymmetric localization of PCP proteins[b]	-1.88	0.011
	Degradation of DVL[b]	-1.80	0.022
Hedgehog signaling	Hedgehog ligand biogenesis disease[b]	-1.80	0.022
	Degradation of GLI1 by the proteasome[b]	-1.79	0.023
	Processing defective Hh variants are degraded by the proteasome[b]	-1.78	0.027
	Hedgehog ligand biogenesis[b]	-1.76	0.033
	Degradation of GLI2 by the proteasome[b]	-1.75	0.033
	GLI3 is processed to GLI3R by the proteasome[b]	-1.72	0.040
Sema4D signaling	Sema4D in semaphorin signaling[b]	2.11	0.040
	Sema4D-induced cell migration and growth cone collapse[b]	2.06	0.038

Pathway source: [a]WikiPathways; [b]Reactome; [c]KEGG; [d]BioCarta.
